# Oseltamivir-Resistant Pandemic (H1N1) 2009 Virus, Mexico

**DOI:** 10.3201/eid1702.100897

**Published:** 2011-02

**Authors:** José Ernesto Ramirez-Gonzalez, Elizabeth Gonzalez-Duran, Patricia Alcantara-Perez, Claudia Wong-Arambula, Hiram Olivera-Diaz, Iliana Cortez-Ortiz, Gisela Barrera-Badillo, Ha Nguyen, Larisa Gubareva, Irma Lopez-Martinez, Jose Alberto Díaz-Quiñonez, Miguel Angel Lezana-Fernández, Hugo Lopez Gatell-Ramírez, Jose Angel Cordova Villalobos, Mauricio Hernández-Avila, Celia Alpuche-Aranda

**Affiliations:** Author affiliations: Instituto de Diagnóstico y Referencia Epidemiológicos, Mexico City, Mexico (J.E. Ramirez-Gonzalez, E. Gonzalez-Duran, P. Alcantara-Perez, C. Wong-Arambula, H. Olivera-Diaz, I. Cortez-Ortiz, G. Barrera-Badillo, I. Lopez-Martinez, J.A. Díaz-Quiñonez, M.A. Lezana-Fernández, H.L. Gatell-Ramírez, J.A.; Cordova Villalobos, M. Hernández-Avila, C. Alpuche-Aranda); Secretaría de Salud, Mexico City (J.E. Ramirez-Gonzalez, E. Gonzalez-Duran, P. Alcantara-Perez, C. Wong-Arambula, H. Olivera-Diaz, I. Cortez-Ortiz, G. Barrera-Badillo, I. Lopez-Martinez, J.A. Díaz-Quiñonez, M.A. Lezana-Fernández, H.L. Gatell-Ramírez, J.A. Cordova Villalobos, M. Hernández-Avila, C. Alpuche-Aranda);; Centers for Disease Control and Prevention, Atlanta, Georgia, USA (H. Nguyen, L. Gubareva)

**Keywords:** Oseltamivir, influenza, neuraminidase, G1N1, pandemic, viruses, Mexico, expedited, dispatch

## Abstract

During May 2009–April 2010, we analyzed 692 samples of pandemic (H1N1) 2009 virus from patients in Mexico. We detected the H275Y substitution of the neuraminidase gene in a specimen from an infant with pandemic (H1N1) 2009 who was treated with oseltamivir. This virus was susceptible to zanamivir and resistant to adamantanes and oseltamivir.

In March and early April 2009, a new strain of influenza A virus that contained genes from the Eurasian–North American triple reassortant and classical swine lineage viruses emerged in North America (*1**,**2*). By May 21, 2010, a total of 214 countries and overseas territories or communities had reported laboratory-confirmed pandemic (H1N1) 2009, which resulted in at least 18,097 deaths in patients with PCR-confirmed illness (*3*). In Mexico 72,533 cases (1,228 deaths) were PCR confirmed by the second week of May 2010 (*4*).

To control influenza A virus infections, the US Food and Drug Administration has approved the use of matrix 2 (M2) blockers, amantadine and rimantadine, and the neuraminidase (NA) inhibitors (NAIs), oseltamivir and zanamivir (*5*). However, for pandemic (H1N1) 2009, therapeutic options are limited to the NAIs because this virus has a swine virus–origin M2 gene, which contains a mutation associated with resistance to adamantanes (*6*). NAI resistance in pandemic (H1N1) 2009 viruses has been rare; nevertheless, 285 oseltamivir-resistant cases were reported worldwide as of April 14, 2010 (*7*). All oseltamivir-resistant viruses have the H275Y substitution that confers resistance to oseltamivir but not to zanamivir. Spread of oseltamivir-resistant seasonal influenza A virus (H1N1) was first detected in 2007, and this virus has now become the predominant lineage of influenza A virus (H1N1) in humans (*8**,**9*). This finding raises strong concerns that the H275Y mutation could become dominant in pandemic (H1N1) 2009 as well. We report oseltamivir-resistant pandemic (H1N1) 2009 detected through virologic surveillance in Mexico.

## The Study

We aimed to determine the drug susceptibility of pandemic (H1N1) 2009 in Mexico. We randomly selected 692 independent clinical samples (452 cell culture supernatants and 199 nasopharyngeal swab specimens [NPS]) or viral isolates, mostly from patients hospitalized in Mexico and from a few symptomatic patients with highly suspected oseltamivir- resistant infections (31 NPS and 10 lung biopsy specimens from patients who died). The study was conducted during July 2009–May 2010. All samples were received during May 2009–April 2010 at the Institute of Epidemiologic Diagnosis and Reference (InDRE [Mexico City, Mexico]); all were positive for pandemic (H1N1) 2009 by real-time reverse transcription–PCR (RT-PCR), according to the procedure recommended by the Centers for Disease Control and Prevention (Atlanta, GA, USA) and the World Health Organization.

Samples were collected from patients in all Mexican states. Patients did not differ significantly by sex, and persons 10–29 years of age were most commonly affected, similar to the number of incident cases of acute respiratory infection in Mexico (Table 1). Viral RNA was extracted by using either MagNA Pure LC Total Nucleic Acid Isolation Kit (Roche Diagnostics, Rotkreuz, Switzerland) or QIAmp Viral RNA Mini Kit (QIAGEN, Hilden, Germany).

**Table 1:** Characteristics of patients with pandemic (H1N1) 2009 reported to the Institute of Epidemiologic Diagnosis and Reference, by age group, Mexico, May 2009–April 2010

Age group, y	No. (%) patients		
	Female	Male	Total
0–1	9	6	15
1–4	30	23	53
5–9	40	34	74
10–19	67	83	150
20–29	66	65	131
30–39	25	34	59
40–49	25	24	49
50–59	19	19	38
>60	5	6	11
Unknown	51	61	112
Total	337 (48.6)	355 (51.3)	692

An endpoint RT-PCR was performed for all 692 samples screened for the H275Y molecular marker by using the Superscript III RT-PCR system (Invitrogen, Carlsbad, CA, USA) and FLUAN1–721F and FLUAN1–924R primers spanning position 275 of the NA gene (Table 2). Direct sequencing of these PCR products was performed by using a sequencing primer (FLUAN1–904R, Table 2) and the BigDye Terminator version 3.1 cycle sequencing reaction kit on an ABI PRISM 3130xl DNA analyzer (Applied Biosystems, Foster City, CA, USA).

**Table 2:** Primer sets used in reverse transcription–PCR and Sanger sequencing of isolates for pandemic (H1N1) 2009, Mexico, May 2009–April 2010*

Primer	Sequence, 5′ → 3′	Target/position, nt
FLUAN1–721F	GTAATGACCGATGGACCAAG	NA/721
FLUAN1–924R	CTGGTTGAAAGACACCCAC	NA/924
FLUAN1–904R	GTCGATTCGAGCCATGCCAG	NA/904
MBTuni-12†	ACGCGTGATCAGCAAAAGCAGG	NA/5′ UTR
MBTuni-13†	ACGCGTGATCAGTAGAAACAAGG	NA/3′ UTR

*NA, neuraminidase; UTR, untranslated region. †Primer sets previously published in 2009 (*11*).

All the sequences obtained indicated that the H→Y mutation at the NA 275 residue was absent, except in 1 NPS from an 8-month-old girl (A/Mexico/InDRE797/2010). This patient received oseltamivir treatment from the evening of January 22 through January 27. She had no known history of travel, contact with a person treated with this drug, or diagnosed immunodeficiency. Her clinical record mentioned 2 respiratory events in the 2 months before the influenza diagnosis: broncholitis, which required hospitalization, and readmission to the hospital 2 weeks after discharge because of influenza-like illness and severe pneumonia. In addition to oseltamivir, the child required other antimicrobial drugs (ceftriaxone and vancomycin) and mechanical ventilation. She slowly recovered.

**Figure 1:**
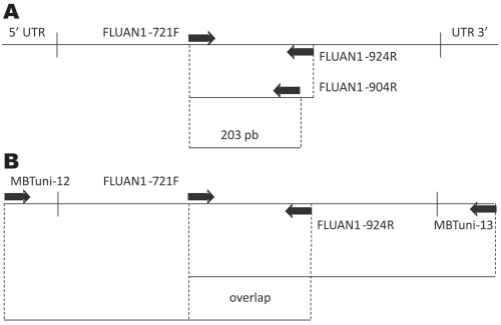
Reverse transcription–PCR (RT-PCR)/sequencing primers scheme for the neuraminidase (NA) gene. A) Primer position for screening RT-PCR protocol. B) Primer position and the 2 overlapping RT-PCR products for the complete NA sequence. UTR, untranslated region.

**Figure 2:**
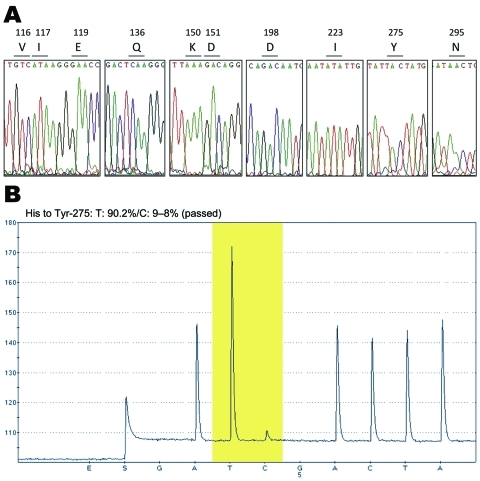
DNA sequence electropherograms for neuraminidase (NA) gene sequences. A) Analysis of molecular markers (V116, I117, E119, Q136, K150, D151, D198, I223, H275, and N295) for oseltamivir and/or zanamivir resistance among the pandemic (H1N1) 2009 virus isolates. The oseltamivir resistance–conferring mutation CAC (histidine) to TAC (tyrosine) at position 275 was detected in the InDRE797 sample. B) Detection of the H275Y mutation in the NA of the viruses by single-nucleotide polymorphism analysis at NA275 position (yellow area).

The clinical specimen was collected, and pandemic (H1N1) 2009 was laboratory confirmed on January 27, 2010. In this sample and in isolating the virus we further analyzed, we discarded any of the 9 previously described mutations (*5**,**10*) associated with resistance to NAIs (V116, I117, E119, Q136, K150, D151, D198, I223, and N295 [N2 numbering]) by the full sequence of the NA gene by using 2 overlapping RT-PCR products (Figure 1, Table 2). The complete NA sequence of the A/Mexico/InDRE797/2009 virus obtained from NPS and MDCK isolate (GenBank accession no. CY057074) confirmed the H275Y substitution and showed no other NA mutations known to be associated with NAI resistance (Figure 2, panel A). Pyrosequencing analysis performed on the clinical specimen from the 8-month-old patient showed oseltamivir-resistant H275Y and wild-type H275 virus variants (Figure 2, panel B). The A/Mexico/InDRE797/2009 virus was tested at the Centers for Disease Control and Prevention by using an NA inhibition assay (NA-Star kit, Applied BioSystems) and showed an ≈120-fold increase in oseltamivir 50% inhibitory concentration over that of a sensitive control (27.3 nmol/L vs. 0.23 nmol/L; this result was consistent with H275Y and H275 variants. No change in zanamivir susceptibility was detected (0.32 nmol/L vs. 0.30 nmol/L), which was in accord with the NA sequencing analysis. We also sequenced the M2 gene and confirmed the S31N substitution that confers M2 blocker resistance. Sequencing of the HA gene showed that the substitution D222G, potentially associated with severe clinical outcome (*12*), was not present in this isolate (data not shown).

In addition, 24 virus isolates collected during December 2009–January 2010 were tested in the NA inhibition assay. All were sensitive to both NAIs, with 50% inhibitory concentrations of 0.12–0.29 nmol/L and 0.17–0.36 nmol/L for oseltamivir and zanamivir, respectively.

No other molecular markers of NAI resistance, such as at residues Q136, K150, or D151, which might confer zanamivir resistance, were observed in the isolate from the 8-month-old girl. The functional NI assay confirmed the oseltamivir resistance and susceptibility to zanamivir of the virus. Both variants (H275Y and wild type) were present in the clinical specimen and its matching virus isolate, which is not unusual (*13*). The proportion of resistant virus (1 [0.14%] of 692 analyzed cases) is lower than has been described, perhaps because of the limited analysis. Analysis of additional samples will enable detection of additional cases. The M2 S31N substitution, the adamantane resistance marker, also was present, as is expected in this virus.

## Conclusions

Concern exists that an oseltamivir-resistant variant of pandemic (H1N1) 2009 virus may emerge and spread in a manner similar to that of oseltamivir-resistant seasonal influenza A virus (H1N1) (*14*). Because pandemic (H1N1) 2009 virus is already resistant to adamantanes, oseltamivir resistance would leave zanamivir as the only antiviral treatment option. Consequently, close monitoring of the antiviral susceptibility of pandemic (H1N1) 2009 strains is critical for controlling the spread of this virus (*15*).
